# Wheat Varietal Response to *Tilletia controversa* J. G. Kühn Using qRT-PCR and Laser Confocal Microscopy

**DOI:** 10.3390/genes12030425

**Published:** 2021-03-16

**Authors:** Delai Chen, Ghulam Muhae-Ud-Din, Taiguo Liu, Wanquan Chen, Changzhong Liu, Li Gao

**Affiliations:** 1 College of Plant Protection, Gansu Agricultural University, Lanzhou 730070, China; cdlbuck@163.com; 2 State Key Laboratory for Biology of Plant Disease and Insect Pests, Institute of Plant Protection, Chinese Academy of Agricultural Sciences, Beijing 100193, China; gm3085pp@outlook.com (G.M.-U.-D.); tgliu@ippcaas.cn (T.L.); wqchen@ippcaas.cn (W.C.)

**Keywords:** wheat dwarf bunt, *TaPR* genes, tapetum, pollen grain, laser confocal microscopy

## Abstract

*Tilletia controversa* J. G. Kühn is a causal organism of dwarf bunt in wheat. Understanding the interaction of wheat and *T. controversa* is of practical and scientific importance for disease control. In this study, the relative expression of *TaLHY* and *TaPR-4* and *TaPR-5* genes was higher in a resistant (Yinong 18) and moderately resistant (Pin 9928) cultivars rather than susceptible (Dongxuan 3) cultivar at 72 h post inoculation (hpi) with *T. controversa*. Similarly, the expression of defensin, *TaPR-2* and *TaPR-10* genes was observed higher in resistant and moderately resistant cultivars after exogenous application of phytohormones, including methyl jasmonate, salicylic acid, and abscisic acid. Laser confocal microscopy was used to track the fungal hyphae in the roots, leaves, and tapetum cells, which of susceptible cultivar were infected harshly by *T. controversa* than moderately resistant and resistant cultivars. There were no fungal hyphae in tapetum cells in susceptible cultivar after methyl jasmonate, salicylic acid and abscisic acid treatments. Moreover, after *T. controversa* infection, the pollen germination was of 80.06, 58.73, and 0.67% in resistant, moderately resistant and susceptible cultivars, respectively. The above results suggested that the use using of resistant cultivar is a good option against the dwarf bunt disease.

## 1. Introduction

Wheat (*Triticum aestivum* L) is one of the most important staple food crops throughout the world. Disease, a main biotic stress, negatively affects plant physiology, morphology, and productivity, and also reduces quality and quantity of wheat worldwide [1]. Dwarf bunt is caused by *T. controversa* and is an economically devastating disease of winter wheat [2]. The disease is a seed and soil-borne and appeared in cold areas of the world [3,4]. The pathogen has the extreme potential to grow when persistent and deep snow occurs before the soil become frozen, which provides a long period of cool, stable, and humid conditions that are suitable for teliospore germination and infection. *T. controversa* is an important quarantine pathogen and many countries have strict restriction for importing wheat grains infected by it [5,6]. The closely related species of *T. controversa* are *T. caries* and *T. foetida*, causing common bunt of wheat, are more widely distributed in the world. *T. caries* and *T. foetida* can be differentiated from *T. controversa* through molecular techniques including internal transcribed spacer (ITS) and intergenic spacers (IGS) [7,8]. Plants and spikes of wheat infected by *T. controversa* are typically shorter than a healthy one [4,9]. In the normal flowering plants, the male reproductive organ stamen usually has four anther lobes; every lobe has microsporagium where pollen grains complete their development. The male reproduction has many steps, including initiation of tapetum cells and generation of germ-line meiotic cells. These tapetum and germ cells support the process of pollen development [10]. The development of functional pollen that is critical to maximize the pollination is important for plant reproduction [10,11,12]. Therefore, the tapetum cells and pollen development are key players for the anther development and pollination. The infected tapetum cells by *Ustilago maydis* increased in size compared to normal cells [12]. The fungal hyphae of *T. controversa* were seen on the somatic and reproductive cells of the wheat anthers [9]. Millions of teliospores of *T. controversa* can develop in the spikelets of wheat [13].

Plants have progressed different mechanisms to manage the biotic stresses [14,15]. Phytohormones, including methyl jasmonate (MeJa), salicylic acid (SA), and abscisic acid (ABA), activate primary defense responses of the plants against both abiotic and biotic stresses via antagonistic or synergistic actions [16]. Usually, MeJa and SA are associated with necrotrophic and biotrophic pathogens, respectively [17]. Whereas, ABA has important role in plant growth and development, and also in defense responses against both biotic and abiotic stresses [18,19]. Transcription factors (TFs) are important molecules in the regulatory networks underlying plant behaviors to biotic and abiotic pressures [20]. The MYB family belongs to TFs and against the plant pathogens [21]. Similarly, pathogenesis-related (PR) proteins have been implicated in defense response, potentially restricting pathogen development and spread [22,23,24]. Both TFs and PR proteins can directly affect pathogen integrity or release signal molecules through their enzymatic activity that act as elicitors molecules to induce other plant defense related pathways [25,26,27,28,29]. *TaLHY* is a 1R protein MYB transcription factor (R1/R2-MYB), which plays critical role in disease resistance against ear heading and stripe rust pathogens of the wheat [30]. Plants have both inducible and performed mechanisms to protest attack of the plant pathogens and respond them by various defense tactics leading to the synthesis of different protective molecules, for example, pathogenesis related proteins (*PR-2* and *PR-5*) [31]. Previous studies showed that there were *PR-1* to *PR-13* proteins families in plants upon infection by fungi, oomycetes, virus, bacteria, nematode, as well as insect attack [32]. The recognized PRs have been broadly reviewed [33,34] and presently have 17 PRs families [22]. Previous studies revealed that *PR-2* and *PR-4* act as the antifungal compounds, which limiting the pathogen growth, activity, and the fitness of fungal plant pathogens [22]. *PR-2* (chitinase) has potential to target the herbivorous and nematodes infection in tomato plants [22]. *Triticum aestivum* pathogenesis related (*TaPR-4*) has antifungal activity against different pathogens and also has ribonuclease activity in wheat [35,36]. Similarly, *RR-10* proteins display homology to ribonucleases, while some members have the weak ribonucleases activity [37]. *PR-5* family has direct link in resistance against oomycetes. Similarly, defensin has comprehensive antifungal and antibacterial activities [22]. The up-regulation or down regulation of *PR-2*, *PR-5* genes increase or decrease the disease severity in wheat and rice [38,39]. 

Previous studies showed that *PR-2*, *PR-5*, and *PR-10* proteins increased resistance level against *Phytophthora infestants*, *Puccinia triticina*, and *Magnaporthe grisea*, in wheat and rice, respectively [24,40,41,42]. Interestingly, *TaLHY*, a wheat 1R-MYB gene had improved tolerance to stripe rust pathogen strain CYR32, silencing analysis suggest that *TaLHY* positively participate in wheat defense response to stripe rust pathogen [30]. The ABA increased the tolerance level in *Arabidopsis thaliana* against *Leptosphaeria maculans* and *Pseudomonas syringae* by induction of *PR-2* genes [43]. Similarly, SA mediated pathways induce the expression of defensin protein against plant pathogens [44].

In potato tubers, qRT-PCR technique was also used to detect successfully *Colletotrichum coccodes* [45]. Using the same technique, the mycelium of *T. caries* and *T. controversa* were quantified in the apical meristem of wheat by using quantitative real-time PCR (qRT-PCR) [46]. Therefore, investigation PR genes expression in wheat cultivars after *T. controversa* infection by using qRT-PCR is very important in the process of plant selection.

In the present study, we checked the presence of *T. controversa* on tapetum cells and pollen grain germination. Furthermore, we investigated the expression of PRs genes (*TaPR-4* and *TaPR-5*) the MYB transcription factor (*TaLHY*) genes and the role of exogenous hormones (MeJa, SA and ABA) in the induction of PRs genes expression (defensin, *TaPR-2*, and *TaPR-10*) in resistant, moderately resistant and susceptible wheat cultivars against dwarf bunt disease. The proliferation of *T. controversa* hyphae was further examined in roots, leaves, and tapetum cells of anther by laser confocal microscopy. The effects of *T. controversa* on pollen grain germination in resistant, moderately resistant and susceptible cultivars were additionally tested.

## 2. Materials and Methods

### 2.1. Plant Material and Fungal Inoculation

In total, three wheat (*Triticum aestivum* L) cultivars (Yinong 18, Pin 9928, and Dongxuan 3) and *T. controversa* were the biological materials of this study. Wheat cultivars were collected from the Institute of Plant Protection, Chinese Academy of Agricultural Sciences, Beijing, China, while *T. controversa* was provided by Blair Goates, National Small Grains Germplasm Research Facility, United States Department of Agriculture-Agricultural Research Service (USDA-ARS). Above cultivars were tested in a greenhouse against *T. controversa* during 2015–2018, Yinong 18, which is known to be very resistant to *T. controversa* (with 5% infected spikes), was used as the resistant cultivar in the present work. Pin 9928, which is known to be very moderately resistant to *T. controversa* (with 27% infected spikes), was used as the moderately resistant cultivar in this study. Dongxuan 3, a very susceptible wheat cultivar to *T. controversa* (73% infected spikes) was used as the susceptible cultivar in the present work. Seeds of above cultivars were grown in the experimental pots in a growth chamber (14 h light: 10 h dark 5 ± 2 °C and 70% relative humidity). A total of four biological replicates of each cultivar were used in this study. The fungal cultivation and inoculation of wheat plants followed the method previously published [9]. Briefly, the concentration of fungal conidia in the ddH_2_O was adjusted to 10^6^ conidia mL^−1^ and inoculated seedlings. Inoculation was repeated five times with one-day interval. The inoculated leaves of above cultivars were sampled at 24, 36, 72, and 96 h post inoculation (hpi), quickly frozen in liquid nitrogen, and stored at −80 °C for further use. The hormone treatments, namely 100 mM of abscisic acid (ABA), 100 mM of methyl jasmonate (MeJa) and 100 mM of salicylic acid (SA) were performed by following our laboratory method [9]. The treated leaves were collected for RNA extraction at 1, 3, and 7 h after hormone treatment. Plants sprayed with ddH_2_O used as a control [9].

### 2.2. RNA Extraction and cDNA Synthesis

Plant samples (100 mg of the leaves) collected for each *T. controversa* infected and control plants were immediately placed in liquid nitrogen and processed for RNA extraction by using EasyPure Plant RNA Kit (TransGen, Beijing, China) following manufacturer instructions. The quality and quantity of extracted RNA were checked through a NanoDrop spectrophotometer (Denovix, Wilmington, DE, USA) device. The RNA was stored at −80 °C until used for cDNA synthesis. First-strand cDNA was synthesized by using 1.5 µg of purified total RNA, RT–RI enzyme and oligo (dT)18 Primer (TransGen) following the instructions of the kit (TransGen) and stored at −20 °C for further use. The cDNA was synthesized from three biological replicates and four technical replicates for qRT-PCR analysis. Additionally, the same RNA extraction and cDNA synthesis method was used for samples treated with MeJa, SA, and ABA at different time intervals.

### 2.3. Quantitative Real-Time PCR Analysis

Quantitative real-time-PCR was performed using SYBR Green Master Mix in a total volume of 20 µL by following the manufactures instructions and applied to the ABI 7500 RT-PCR system (Applied Biosystems, Foster City, CA, USA). The qRT-PCR reactions were set up with the following thermal cycles: pre-denaturation at 95 °C for 10 min and 40 cycles of 95 °C for 15 s, 58 °C for 30 s, and 72 °C for 30 s. The amplification of wheat actin gene was used as an internal control for normalizing all data. The 2^−ΔΔCT^ method [47] was used to calculate the relative expression of every gene. The genome of wheat crop is complex when compared with other crops due to its hexaploid nature. The interaction between the three subgenomes subsidize flexibility in gene expression levels, which enhanced the adaptability to various biotic and abiotic factors [48,49]. The primers used in this study are positioned in an identical region to the three subgenomes of wheat and listed in Table S1.

### 2.4. Observation by Laser Confocal Microscopy

Roots, leaves, and anther cells were investigated under laser confocal microscopy to investigate the fungal intensity in resistant, moderately resistant, and susceptible cultivars, as previously described [2,9]. Briefly, the roots, leaves and anthers were dissected from the wheat and immediately dip in absolute ethanol (96%) until the tissues changed into white. The anther cells were stained with Propidium Iodide (PI) (Invitrogen, Eugene, OR, USA) and fungal hyphae in the roots, leaves and tapetum cells were stained with the chitin-specific dye Wheat Germ Agglutinin and Alexa Flour 488 conjugate (WGA-AF488) (Invitrogen). After 1 h slides were made and investigated under laser confocal microscopy (Leica SP8, Wetzlar, Germany), as described before [50].

### 2.5. Effects of T. controversa on Pollen Germination

The mature anthers with stamen were collected from mock and fungal inoculated plants for pollen germination test. Three anthers were collected and gently shaken in 1.5 mL centrifuge tube containing liquid culture media (20% sucrose, 20% PEG4000, 40 mg/L H_3_BO_3_, 3 × 10^−3^ mol/L Ca (NO_3_)_2_ and 10 mg/L VB1) for taking pollen out from locule with slight modification [51]. These centrifuge tubes were incubated at 28 °C for 30, 60 and 90 min time intervals. One drop of every time interval sample was observed under microscope (Leica DM 2500, Wetzlar, Germany). The size of pollen tube half or more from pollen diameter was considered the standard to indicate the ability of the pollen to germinate. Pollen germination was calculated as:

(1) $$\mathrm{pollen}\;\mathrm{germination}=\frac{\mathrm{number}\;\mathrm{of}\;\mathrm{germinated}\;\mathrm{pollen}\;\mathrm{grains}}{\mathrm{total}\;\mathrm{number}\;\mathrm{of}\;\mathrm{observed}\;\mathrm{pollen}\;\mathrm{grain}}\;\times 100$$

### 2.6. Assessment of Wheat Cultivars Against T. controversa

A total of 45 heads of above cultivars were evaluated in response to *T. controversa* for disease assessment. The score for dwarf bunt is as follows

(2) $$\mathrm{Dwarf}\;\mathrm{bunt}=\frac{\mathrm{number}\;\mathrm{of}\;\mathrm{infected}\;\mathrm{heads}}{\mathrm{total}\;\mathrm{number}\;\mathrm{of}\;\mathrm{heads}}\times 100$$

The level of disease resistance was calculated by following the scale mention in our previous study [9]. 

### 2.7. Statistical Analysis

Data were statistically analyzed using one-way (ANOVA) followed by Tukey’s test in SPSS Statistics software (Version 20.0). The results were considered significant at the 5% probability level (*p* ≤ 0.05). The standard errors were calculated in Excel 2016 (Microsoft, Redmond, WA, USA).

## 3. Results

### 3.1. The Expression Patterns of TaLHY, TaPR-4, and TaPR-5 in Response to T. controversa Infection

**Figure 1:**
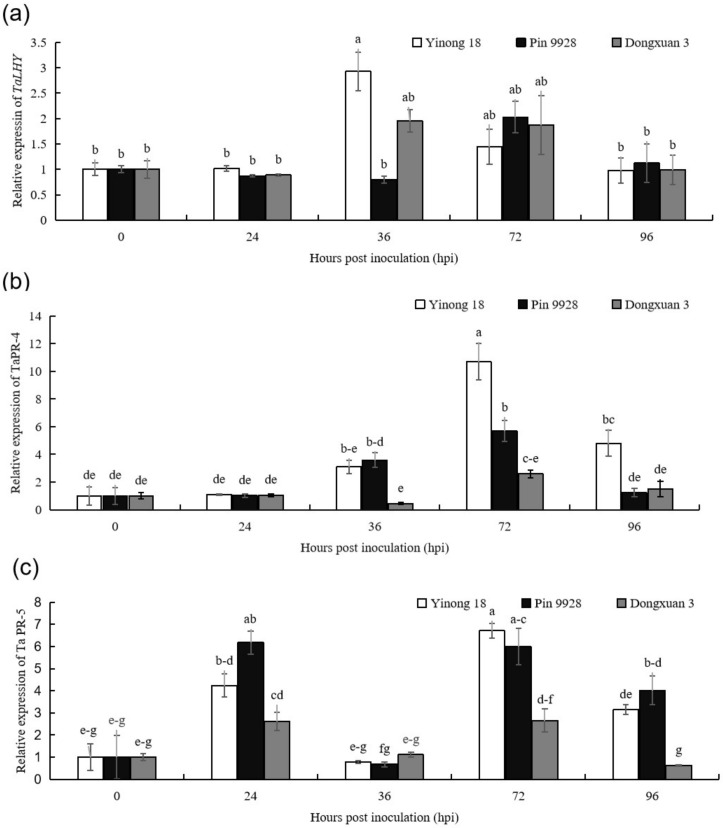
Expression profiles of *TaLHY*, *TaPR-4*, and *TaPR-5* in resistant (Yinong 18), moderately resistant (Pin 9928), and susceptible (Dongxuan 3) wheat cultivars at different time intervals after *T. controversa* infection. (**a**) relative expression of *TaLHY;* (**b**) relative expression of *TaPR-4;* (**c**) relative expression of *TaPR-5*. The *T. controversa* treatment at every time point is normalized at 0 hpi. The significant differences were statistically analyzed based on three biological replications and four technical replications (Tukey’s test: *p* < 0.05). Bars indicate the standard errors. Lettering above the bars showed level of significance.

The relative expression value was measured in leaves in resistant (Yinong 18), moderately resistant (Pin 9928) and susceptible (Dongxuan 3) wheat cultivars by using qRT-PCR. The results showed that at 36 h post inoculation (hpi), the relative expression of *TaLHY* in resistance cultivar was significantly up-regulated compared to moderately resistant and susceptible cultivars by comparing the expression at 0 hpi (control) (*p* < 0.05), the expression of which increased to 2.28-fold. The relative expression was statistically significant at 72 hpi for above tested cultivars (Figure 1A). As shown in Figure 1B, results revealed that transcripts abundance of *TaPR-4* protein was statistically high in resistant cultivar at 72 hpi compared with expression at 0 hpi (*p* < 0.05), which was 10.71-fold of the relative expression at 0 hpi, and also higher than that in the moderately resistant and susceptible wheat cultivars at the corresponding time (*p* < 0.05) (Figure 1B). Similarly, relative expression of *TaPR-5* protein was statistically high (6.71-fold) at 72 hpi in resistant cultivar, which was followed by the moderately resistant cultivar (6.18-fold) at 24 hpi compared with the expression at 0 hpi (*p* < 0.05). Additionally, the relatively expression of *TaPR-5* in moderately resistant cultivar was significantly up-regulated at 72 and 96 hpi, compared with expression at 0 hpi (*p* < 0.05), which was 5.99-fold and 4.02-fold, respectively. Values found were significantly higher than that in the susceptible cultivar at the corresponding time (Figure 1C). The results clearly revealed that the expression of *TaLHY, TaPR-4*, and *TaPR-5* were higher in resistant and moderately resistant wheat cultivars, which can positively indicate that these genes possibly regulate resistant in the resistance and moderately resistant cultivars. Therefore, using of resistance cultivars is the best option against the dwarf bunt pathogen.

### 3.2. Response of Pathogenesis Related Proteins against to Exogenous Hormones in Different Wheat Cultivars

**Figure 2:**
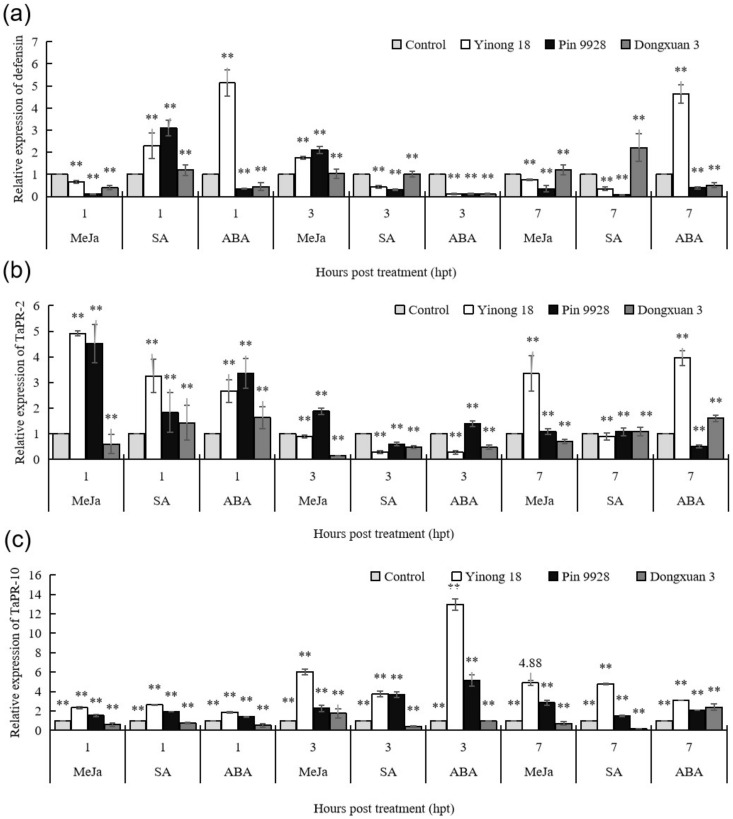
Transcriptional patterns of defensin, *TaPR-2* and *TaPR-10* after treatment with hormones. The resistant (Yinong 18), moderately resistant (Pin 9928) and susceptible (Dongxuan 3) wheat cultivars were sprayed with Methyl jasmonate (MeJa), salicylic acid (SA), and abscisic acid (ABA). The plants treated with ddH_2_O were used as control in the study. (**a**) relative expression of defensin; (**b**) relative expression of *TaPR-2;* (**c**) relative expression of *TaPR-10*. The significant differences were statistically analyzed based on three biological replications and four technical replications (Tukey’s test: ** *p* < 0.05). Bars indicate the standard errors.

Transcriptional profiles of defensin, *TaPR-2* and *TaPR-10* were analyzed by qRT-PCR in resistant (Yinong 18), moderately resistant (Pin 9928) and susceptible (Dongxuan 3) wheat cultivars after exogenous hormone treatment; including MeJa, SA, and ABA at 1, 3, and 7 h post treatment (hpt). Leaves of the above cultivars during jointing stage were treated with hormones and relative expression was measured at 3 times point; namely 1, 3, and 7 hpt. In Figure 2A, for defensin, ABA induced the maximum level of relative expression at 1 and 7 h post treatment (hpt) with a 5.15-fold and 4.63-fold increase in resistant cultivar compared to control, respectively. However, ABA treatment downregulated the expression of defensin by 0.12-fold (resistant), 0.12-fold (moderately resistant), and 0.11-fold at 3 hpt compared to the control. With regard to SA, the highest relative expression of defensin was noted at 1 and 7 hpt, reaching a 2.29-fold and 2.21-fold increase, respectively, compared to control. In the case of MeJa, the maximum transcriptional level of defensin was noted at 3 hpt, reaching a 2.09-fold increase compared to the control in moderately resistance cultivar. 

In Figure 2B, the response of *TaPR-2* protein to the exogenous application of hormones was comparatively higher at 1 hpt than 3 and 7 hpt compared to the control. The expression level of *TaPR-2* increased to 4.91-fold (MeJa) and 4.51-fold (SA) at 1 hpt in resistant and moderately resistant cultivars, respectively, compared to control. Similarly, *TaPR-2* responded in a similar way to the exogenous application of SA and ABA at 1 hpt in resistant and moderately resistant cultivars. The highest expression in the SA occurred at 1 hpt with an increase of 3.25-fold in resistant cultivar and in the ABA, expression increased to 3.35-fold. At 3 hpt, *TaPR-2* expression was decreased by 0.89-fold and 0.14-fold in resistant and susceptible cultivars, respectively, in the case of MeJa compared to control. The expression increased to 1.87-fold in the moderately resistant cultivar at 3 hpt for the MeJa. After the SA treatment, *TaPR-2* expression was decreased by 0.28-fold, 0.59-fold, and 0.48-fold in resistant, moderately resistant, and susceptible cultivars, respectively, compared to control at 3 hpt. Similarly, after the ABA treatment, *TaPR-2* expression was decreased by 0.27-fold and 0.47-fold in resistant and susceptible cultivars, respectively, compared to reference at 3 hpt. In the 7 hpt MeJa treatment, the expression level of *TaPR-2* was 3.34-fold higher in the resistant cultivar compared to control. Similarly, for the ABA, expression level of *TaPR-2* was 3.96-fold higher in the resistant cultivar compared to control.

As shown in Figure 2C, the *TaPR-10* expression levels after treatments for 1, 3, and 7 hpt for resistant, moderately resistant and susceptible cultivars were analyzed. In the resistant cultivar, after the treatment with MeJa, the *TaPR-10* expression levels at the 1, 3, and 7 hpt were increased by 2.33-fold, 6.00-fold, and 4.88-fold, respectively, compared to control. Using the same hormonal compound in moderately resistant cultivar, the *TaPR-10* expression levels at the 3 and 7 hpt were increased by 2.27-fold and 2.83-fold, respectively, compared to control. However, the expression levels of susceptible cultivar at 1 and 7 hpt decreased by 0.59-fold and 0.71-fold, respectively, after the MeJa treatment. Applying SA treatment in the resistant cultivar, the *TaPR-10* expression levels at the 1, 3, and 7 hpt increased by 2.62-fold, 3.74-fold, and 4.77-fold, respectively, compared to control. For the moderately resistant cultivar, and after application of SA treatment, the *TaPR-10* expression level at the 3 hpt was increased by 3.66-fold compared to control. However, applying SA treatment in the susceptible cultivar, the *TaPR-10* expression levels at 1, 3, and 7 hpt decreased by 0.75-fold, 0.41-fold, and 0.15-fold, respectively, compared to control. For the ABA treatment for resistance cultivar, the *TaPR-10* expression levels at 3 and 7 hpt was increased by 12.96-fold and 3.07-fold, respectively, compared to control. While for the moderately resistance cultivar, expression levels of *TaPR-10* increased to 5.15-fold and 2.06-fold at 3 and 7 hpt, respectively. However, expression levels decreased by 0.51-fold and 0.96-fold for the susceptible cultivar at 1 and 3 hpt, respectively.

### 3.3. Proliferation of Fungal Hyphae in Root and Leaf Cells

**Figure 3:**
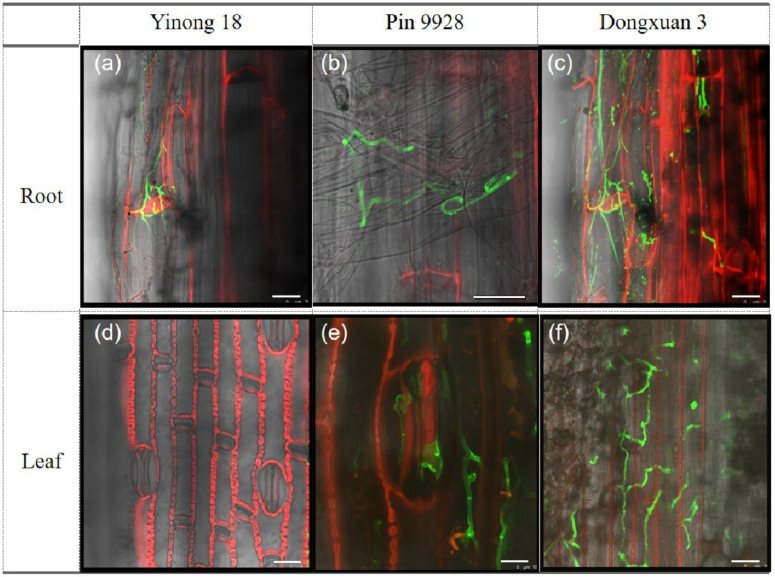
Infestation of *T. controversa* in wheat roots and leaves as indicated by staining with WGA-AF 488 (for hyphae) and propidium iodide (for roots and leaves cell) (**a**) severity of fungal hyphae in roots of resistant (Yinong 18) cultivar; (**b**) severity of fungal hyphae in roots of moderately resistant (Pin 9928) cultivar; (**c**) severity of fungal hyphae in roots of susceptible (Dongxuan 3) cultivar; (**d**) severity of fungal hyphae in leaves of resistant (Yinong 18) cultivar; (**e**) severity of fungal hyphae in leaves of moderately resistant (Pin 9928) cultivar; (**f**) severity of fungal hyphae in leaves of susceptible (Dongxuan 3) cultivar. Scale bar = 25 µm.

To track the hyphae in roots and leaves of resistant, moderately resistant and susceptible wheat cultivars, roots and leaves samples were analyzed by laser confocal microscopy. At germination, hyphae started from small tips, and they formed a hyphal network inside the cortical and rhizodermal cells of roots and leaves. Hyphae moved into cortical and rhizodermal cells through intercellular spaces where they branched and continued to grow. Results revealed that cortical and rhizodermal cells of roots and leaves of susceptible cultivar were harshly infected compared to resistance and moderately resistance cultivars (Figure 3A–C). Similar response was noted in the leaves tissue of above cultivars (Figure 3D–F). 

### 3.4. Proliferation of Fungal Hyphae in Anther Cells

**Figure 4:**
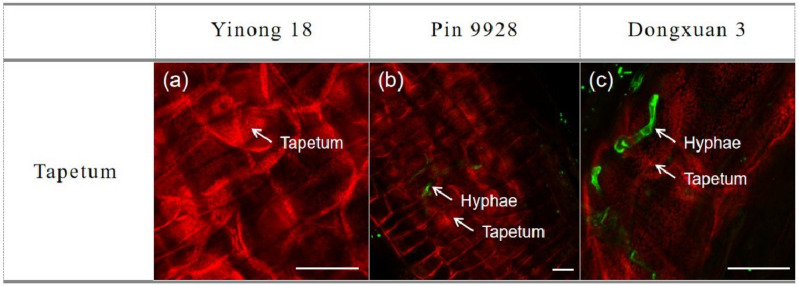
Examination of fungal hyphae in tapetum cells of anther in resistant (Yinong 18), moderately resistant (Pin 9928) and susceptible (Dongxuan 3) wheat cultivars. (**a**) examination of fungal hyphae in tapetum cells of resistant cultivar; (**b**) examination of fungal hyphae in tapetum cells of moderately resistant cultivar; (**c**) examination of fungal hyphae in tapetum cells of susceptible cultivar. Scale bar = 25 µm.

We also observed the proliferation and colonization of fungal hyphae in tapetum cells of anther. The laser confocal microscopy results showed that there was no proliferation and colonization of fungal hyphae into the tapetum cells of resistant cultivar. Moreover, very few hyphae were observed in the tapetum cells of moderately resistant cultivar, but tapetum cells of susceptible cultivar were harshly infected by fungal hyphae (Figure 4A–C). Additionally, there was no fungal hyphae on epidermis and endothecium cells of anther in resistant cultivar. Yet, in the moderately resistance cultivar only a few hyphae were seen, but epidermis and endothecium cells of anther were heavily infected by fungal hyphae (Figure S1).

### 3.5. Effects of Exogenous Hormones on Tapetum Cells of Anther

**Figure 5:**
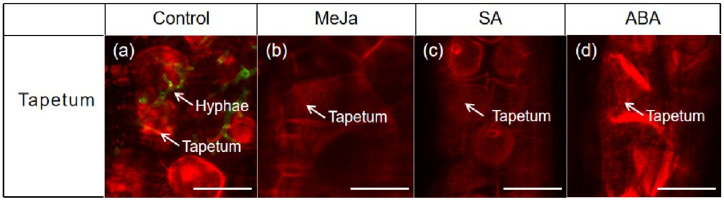
Effect of exogenous hormones on tapetum cells of anther in susceptible cultivar (Dongxuan 3). (**a**) hyphae were located on the tapetum cells in control anthers; (**b**) There were no hyphae on tapetum cells of anther in MeJa treated samples; (**c**) There were no hyphae on tapetum cells of anther in SA treated samples; (**d**) There were no hyphae on tapetum cells of anther in ABA treated samples. Scale bar = 25 µm.

To track the fungal hyphae in the tapetum cells of anther of susceptible cultivar after the treatment of cultivars with MeJa, SA, and ABA were analyzed using laser confocal microscopy. No fungal hyphae were observed in the tapetum cells, which were treated with MeJa, SA, and ABA hormones, but heavily infection of fungal hyphae was observed in the tapetum cells of controls (Figure 5).

### 3.6. Effects of T. controversa on Pollen Grain Germination

**Figure 6:**
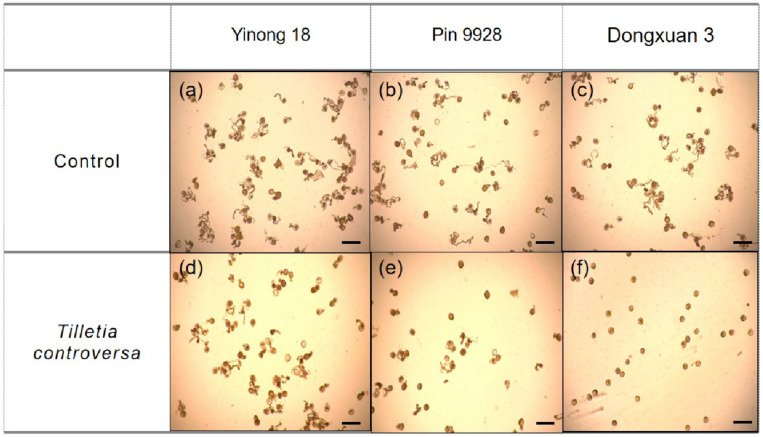
Effect of *T. controversa* on pollen germination (%). (**a**) pollen germination in control samples of resistant cultivar (Yinong 18); (**b**) pollen germination in control samples of moderately resistant cultivar (Pin 9928); (**c**) pollen germination in control samples of susceptible cultivar (Dongxuan 3); (**d**) pollen germination in *T. controversa* infected samples of resistant cultivar (Yinong 18); (**e**) pollen germination in *T. controversa* infected samples of moderately resistant cultivar (Pin 9928); (**f**) pollen germination in *T. controversa* infected samples of susceptible cultivar (Dongxuan 3) Scale bar = 100 µm.

We examined the effects of *T. controversa* in pollen grain germination in vitro. Pollen germination in control was 87.14, 88.39, and 86.95%, while in *T. controversa* infected was 80.06, 58.73, and 0.67% in resistant, moderately resistant and susceptible cultivars, respectively (Table 1 and Figure 6).

### 3.7. Evaluation of Dwarf Bunt Resistance in Wheat Cultivars

**Figure 7:**
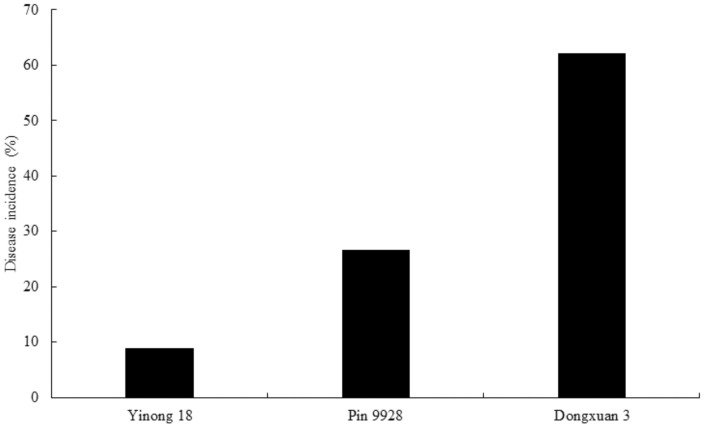
Level of disease incidence in resistant (Yinong 18), moderately resistant (Pin 9928) and susceptible (Dongxuan 3) wheat cultivars to *T. controversa* infection. Yinong 18 showed 8.89%, Pin 9928 showed 26.67%, and Dongxuan 3 showed 62.2% disease incidence.

The resistant (Yinong 18), moderately resistant (Pin 9928) and susceptible (Dongxuan 3) cultivars were evaluated for disease resistance, which showed 8.89, 26.67, and 62.2% infected heads by dwarf bunt pathogen, respectively (Figure 7). This level of infection confirmed that Yinong 18, Pin 9928, and Dongxuan 3 are resistant, moderately resistant, and susceptible cultivars [52]. Additionally, the dwarf bunt symptoms were clearly seen on the spike of susceptible compared to moderately resistant and resistant cultivars (Figure S2).

## 4. Discussion

The qRT-PCR is a highly reliable, sensitive, accurate, and simple method to quantify the expression levels of genes in crops including; wheat after pathogen infection [9,46], while, laser confocal microscopy helps to visualize the fungal and plant cells by using dyes. Previously, Wheat Germ Agglutinin and Alexa Flour 488 conjugate (WGA-AF488) (Invitrogen, Eugene, OR, USA) was used for fungal hyphae and Propidium Iodide (PI) (Invitrogen, Eugene, OR, USA) for plant cell counting [9,53]. Here, we report the expression profiles of pathogenesis-related genes and the infection process of fungal hyphae in the tapetum cells of anther in the resistance, moderately resistance and susceptible cultivars by using qRT-PCR and laser confocal microscopy, respectively. PR proteins of wheat, tomato, and *Arabidopsis* contain a group of functionally and inducible diverse proteins that are accumulated in response to pathogen infection. These proteins have been implicated in active defense, as well as potentially restricting pathogen spread and development [9,22,54,55,56,57]. Regarding the role of wheat and rice PRs proteins in defense system, PR-2, PR-5, and PR-10 proteins can directly affect pathogen integrity or release signal molecules through their enzymatic activity that act as elicitors to induce plant defense related pathways [22,23,24]. Endochitinases (*PR-4*) and thaumatin like proteins (*PR-5*) of wheat, maize, barley, sorghum, and oat are implicated in defense responses against a diverse group of pathogens, including fungal and oomycete pathogens with different lifestyle [22,24,58,59]. Similarly, MYB transcription factors (*TaLHY*) plays key roles in defense mechanism of the plants [30,60]. Previous studies showed that *TaLHY* plays key role in disease resistance against stripe rust of wheat [30]. The expression of PRs genes up-regulated in the resistance cultivar than in susceptible wheat cultivar upon infection by *Bipolaris sorokiniana* and *T. controversa* [9,31]. The silencing or overexpression of *TaPR-5* and *TaLHY* genes decrease or increase the resistance level against plant pathogens [30,61,62]. Here in the qRT-PCR analysis results revealed that infection with *T. controversa* triggers the expression levels of PRs genes (*TaPR-4* and *TaPR-5*) and *TaLHY* gene more in resistant and moderately resistance cultivars than in the susceptible cultivar. After the infection of *T. controversa*, the expression levels of *TaLHY*, *TaPR-4*, and *TaPR-5* were higher in resistant and moderately resistant cultivars than in susceptible cultivar at different time points (Figure 1A–C). The above results revealed that *TaLHY*, *TaPR-4*, and *TaPR-5* genes were activated upon infection by *T. controversa*.

MeJa, SA, and ABA are involved in both biotic and abiotic stress signaling in plants [2,16,17,18] and many defense related genes are activated by MeJa, SA, and ABA [16,17,18,57]. According to the previous literature, the expression of *TaPRs* genes against *B. sorokiniana*, *T. controversa*, and *P. striiformis f.sp. tritici* were increased by above molecules [2,9,30,63]. Our results showed that response of MeJa, SA, and ABA to the expression of *TaPRs* (defensin, *TaPR-2*, and *TaPR-10)* genes was higher at different time points in resistant and moderately resistant cultivars than in the susceptible cultivars. However, the expression induced by MeJa and SA was greater than ABA in above mentioned cultivars (Figure 2A–C).

The Red Bobs a winter wheat cultivar was shown to be more susceptible to dwarf bunt typically during the 1 to 3 leaf stages [64]. The fungal hyphae that established in the 1 to 3 leaf stages remains sparse until reached to the reproductive organs [65]. In present study, we investigated the varietal response to study the proliferation of *T. controversa* on roots, leaves, and tapetum cell of the anthers. Results showed that roots and leaves of susceptible cultivar had harshly infected rather than resistance and moderately resistance cultivars. The fungal hyphae move from roots to reproductive parts as crop mature in susceptible cultivar and further infect the anther cells. The anthers have four lobes that are designed to produce and release pollen grains. Every lobe has a specialized chamber known as locule in which pollen develops. Locule walls are lined by a specialized tissue which are composed by the tapetum cells. The tapetum cell is the innermost layer of the anther and provides nutrients to developing pollen grains. The tapetum cells undergoes to program cell death by depositing a mixture of wax and protein on the surface of pollen exine during the later stages of pollen development [66,67]. *Ustilago maydis* deforms the four anther lobes which influence the normal process of pollen grain development [12]. Our previous studies revealed that hyphae of *T. controversa* were present on the anther epidermal and sub-epidermal cells including; epidermis cells (EPI), endothecium cells (EN), middle layer (ML), and pollen mother cells (PMC) more severely in susceptible cultivars [2,9]. However, here, we observed the prevalence of *T. controversa* in tapetum cells in resistant, moderately resistant, and susceptible cultivars. The results of this study revealed that tapetum cells of susceptible cultivar was harshly infected by fungal hyphae than moderately resistant and resistant cultivar (Figure 4). Additionally, we also confirmed that the percentage of pollen germination was statistically lower in the susceptible cultivar rather than resistance (Table 1 and Figure 5). The pollination from infected anthers is critical for normal plant reproduction. The seeds produced from infected anthers contain millions of teliospores, which turn the grain materials into black mass of *T. controversa* teliospores [4].

## Figures and Tables

**Table 1:** (**a**) Germination (%) of wheat pollen in control and under *T. controversa* infection in resistance (Yinong 18), moderately resistance (Pin 9928) and susceptible (Dongxuan 3) wheat cultivars; (**b**). *t*-test of germination (%) of wheat pollen grains.

	Yinong 18		Pin 9928		Dongxuan 3	
Germination (%)	Control	*T. controversa*	Control	*T. controversa*	Control	*T. controversa*
**(a)**	87.14 ± 2.16	80.06 ± 3.09	88.39 ± 1.57	58.73 ± 2.56	86.95 ± 3.60	0.67 ± 0.38
**(b)**	0.0003 *	0.0004 *	0.0005 *	0.0004 *	0.0003 *	0.0003 *

A total of three replications were used for every variety and more than 200 pollens were used in every replication. * stands for highly significant and ± represents the standard error between replications.

## Supplementary Materials

The following are available online at https://www.mdpi.com/2073-4425/12/3/425/s1, Figure S1: *T. controversa* hyphae is on the epidermal and endothecium cells in resistant, moderately resistant and susceptible cultivars. WGA-AF488 appeared green in hyphae, while PI appeared red in anther cells. (**a**) there is no fungal hyphae on epidermal cells in resistant cultivar (Yinong 18). (**b**) fungal hyphae on epidermal cells in moderately resistant cultivar (Pin 9928) (**c**) fungal hyphae on epidermal cells in susceptible cultivar (Dongxuan 3). (**d**) there is no fungal hyphae on endothecium cells in resistant cultivar (Yinong 18). (**e**) fungal hyphae on endothecium cells in moderately resistant cultivar (Pin 9928). (**f**) fungal hyphae on endothecium cells in susceptible cultivar (Dongxuan 3). Figure S2: Symptoms on the spike of resistant (Yinong 18), moderately resistant (Pin 9928) and susceptible (Dongxuan 3) cultivars at the ripening stage. (**a**) symptoms on resistant (Yinong 18) cultivar (**b**) symptoms on moderately resistant (Pin 9928) cultivar (**c**) symptoms on susceptible (Dongxuan 3) cultivar. The yellow marks showed the bunt sori in the spike. Table S1: List of primers.
