# Supplementary material for: Wheat Varietal Response to Tilletia controversa J. G. Kühn Using qRT-PCR and Laser Confocal Microscopy
Source: Genes (Basel). 2021 Mar 16;12(3):425. doi: 10.3390/genes12030425 (PMC8000713; doi:10.3390/genes12030425)
Supplement: Supplementary file 1 [file genes-12-00425-s001.pdf]

genes

MDPI

Article

Wheat varietal response to *Tilletia controversa* J. G. Kühn using qRT-PCR and laser confocal microscopy

Delai Chen[1,2], Ghulam Muhae-Ud-Din[2], Taiguo Liu[2], Wanquan Chen[2], Changzhong Liu[1,*] and Li Gao[2*]

Supplementary Materials

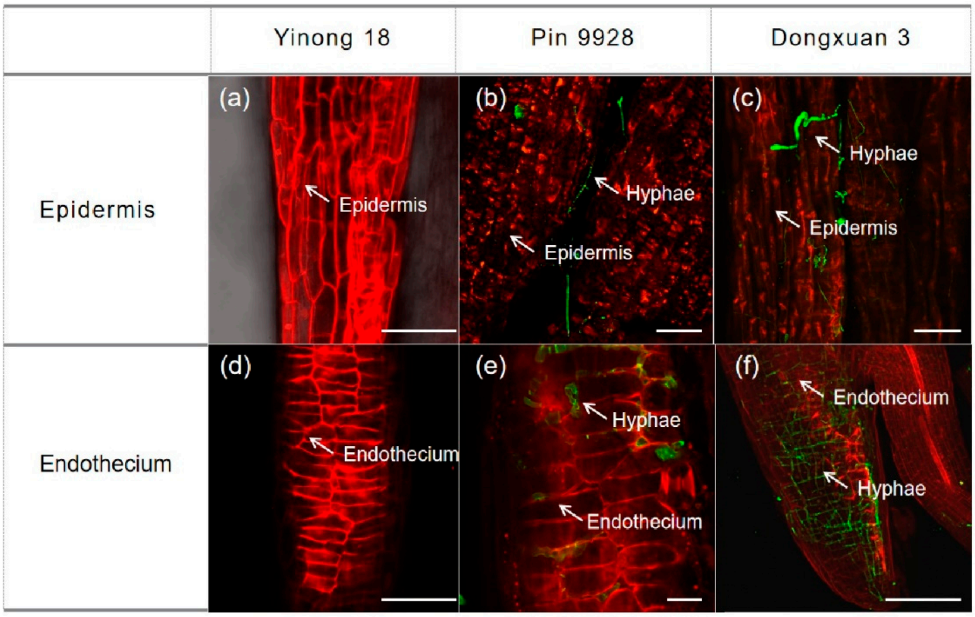

Figure 1. *T. controversa* hyphae is on the epidermal and endothecium cells in resistant, moderately resistant and susceptible cultivars. WGA-AF488 appeared green in hyphae, while PI appeared red in anther cells. (**a**) there is no fungal hyphae on epidermal cells in resistant cultivar (Yinong 18). (**b**) fungal hyphae on epidermal cells in moderately resistant cultivar (Pin 9928) (**c**) fungal hyphae on epidermal cells in susceptible cultivar (Dongxuan 3). (**d**) there is no fungal hyphae on endothecium cells in resistant cultivar (Yinong 18). (**e**) fungal hyphae on endothecium cells in moderately resistant cultivar (Pin 9928). (**f**) fungal hyphae on endothecium cells in susceptible cultivar (Dongxuan 3).

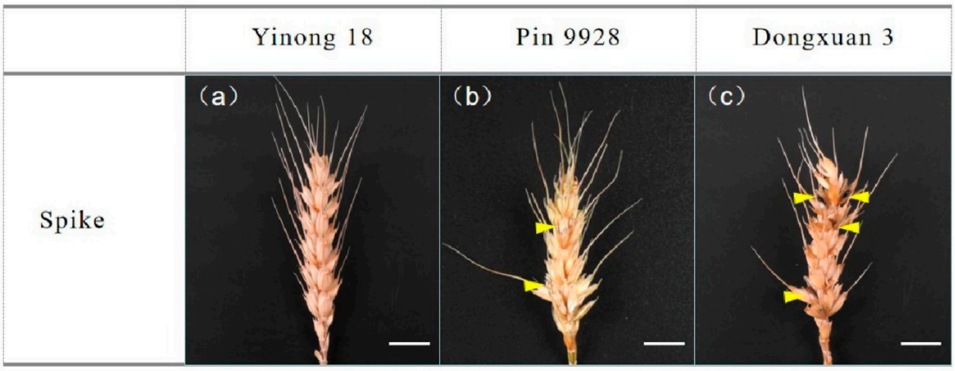

Figure 2. Symptoms on the spike of resistant (Yinong 18), moderately resistant (Pin 9928) and susceptible (Dongxuan 3) cultivars at the ripening stage. (a) symptoms on resistant (Yinong 18) cultivar (b) symptoms on moderately resistant (Pin 9928) cultivar (c) symptoms on susceptible (Dongxuan 3) cultivar. The yellow marks showed the bunt sori in the spike.

Table 1. Sequences of primers used in this study.

Primer Name	Sequence (5′-3′)	Usage
TaLHY F	5′-cctactgcttcctttcccacaac -3′	qRT-PCR
TaLHY R	5′-ctctccttttccactctcgtctg -3′	
PR10 F	5′-cgtggaggtaaacgatgag -3′	qRT-PCR
PR10 R	5′-gctaagtgtccggggtaat -3′	
Defensin F	5′-atgtccgtgccttttgcta -3′	qRT-PCR
Defensin R	5′-ccaaactaccgagtccccg -3′	
PR 4 F	5′-cgaggatcgtggaccagtg -3′	qRT-PCR
PR 4 R	5′-gtcgacgaactggtagttgacg -3′	
PR 2 F	5′-ccgcacaagacacctcaagata -3′	qRT-PCR
PR 2 R	5′-cgatgcccttggtttggtaga-3′	
PR 5 F	5′-acagctacgccaaggacgac -3′	qRT-PCR
PR 5 R	5′-cgcgtcctaatctaagggcag-3′	
Actin F	5′-cactggaatggtcaaggctg -3′	qRT-PCR
Actin R	5′-ctccatgtcatcccagttg -3′	
